# Implementation of 100% smoke-free law in Uganda: a qualitative study exploring civil society’s perspective

**DOI:** 10.1186/s12889-018-5869-8

**Published:** 2018-07-28

**Authors:** Lindsay Robertson, Kellen Namusisi Nyamurungi, Shannon Gravely, Jean Christophe Rusatira, Adeniyi Oginni, Steven Ndugwa Kabwama, Achiri Elvis Ndikum, Eduardo Bianco, Salim Yusuf, Mark D. Huffman

**Affiliations:** 1 Department of Preventive and Social Medicine, University of Otago, PO Box 56, Dunedin, 9054 New Zealand; 2 Centre for Tobacco Control in Africa, Kampala, Uganda; 3 International Tobacco Control Policy Evaluation Project, University of Waterloo, Waterloo, Canada; 4 Johns Hopkins Bloomberg School of Public Health, Baltimore, USA; 5 Nigerian Heart Foundation, Lagos, Nigeria; 6 Mental Health and Substance Abuse, Ministry of Health, Kampala, Uganda; 7 Framework Convention Alliance (AFRO region), Yaoundé, Cameroon; 8 Centro de Investigacion para la Epidemia del Tabaquismo, Montevideo, Uruguay; 9 McMaster University, Hamilton, Canada; 10 Northwestern University, Chicago, USA

**Keywords:** Tobacco control, Smoke-free, Second-hand smoke, Qualitative, Global health, Policy implementation

## Abstract

**Background:**

In 2016, Uganda became one of few sub-Saharan African countries to implement comprehensive national smoke-free legislation. Since the World Health Organisation recommends Civil Society Organisation’s (CSO) involvement to support compliance with smoke-free laws, we explored CSOs’ perceptions of law implementation in Kampala, Uganda, and the challenges and opportunities for achieving compliance. Since hospitality workers tend to have the greatest level of exposure to second-hand smoke, we focussed on implementation in respect to hospitality venues (bars/pubs and restaurants).

**Methods:**

In August 2016, three months after law implementation, we invited key Kampala-based CSOs to participate in face-to-face semi-structured interviews. Interviews probed participants’ perceptions about law implementation, barriers impeding compliance, opportunities to enhance compliance, and the role of CSOs in supporting law implementation. Interviews were recorded and transcribed. Qualitative content analysis was conducted using the interview transcripts.

**Results:**

Fourteen individuals, comprising mainly senior managers from CSOs, participated and reported poor compliance with the smoke-free law in hospitality venues. Respondents noted that contributing factors included low awareness of the law amongst the general public and hospitality staff, limited implementation activities due to scarce resources and lack of coordinated enforcement. Opportunities for improving compliance included capacity building for enforcement agency staff, routine monitoring, rigorous enactment of penalties, and education about the smoke-free law aimed at hospitality venue staff and the general public. Allegations of tobacco industry misinformation were said to have undermined compliance. Civil Society Organisations saw their role as supporting law implementation through education, stakeholder engagement, and evidence-based advocacy.

**Conclusions:**

This study suggests that the process of smoke-free law implementation in Uganda has not aligned with World Health Organisation (WHO) guidelines for implementing smoke-free laws, and highlights that low-income countries may need additional support to enable them to effectively plan for policy implementation and resist industry interference.

## Background

Exposure to second-hand smoke (SHS) from tobacco causes a multitude of harms, including lung cancer, heart disease, respiratory illnesses and increased risk of sudden infant death syndrome among children [1, 2]. Globally, around 890,000 deaths each year result from non-smokers being exposed to SHS [2]. Because there is no safe level of SHS exposure, smoke-free laws mandated by Article 8 of the World Health Organization (WHO) Framework Convention on Tobacco Control (FCTC; [3]) are one of the most important tobacco control measures available. Effective implementation of comprehensive smoke-free laws substantially reduces or eliminates SHS in public places, thereby reducing tobacco-related illnesses and hospitalizations amongst non-smokers [4, 5], helping smokers to quit [6], and preventing smoking initiation [7]. Further, smoke-free workplaces are associated with increased worker productivity and do not adversely impact businesses such as bars and restaurants; in fact, they may lead to marginally better financial or employment outcomes [8].

Arguably, legislation to create smoke-free workplaces and public places is the tobacco control measure that has seen the greatest progress in terms of the extent of policy adoption. At the end of 2014, 49 countries, including 34 low- and middle-income countries (LMICs), had implemented national comprehensive smoke-free legislation [8]. This has resulted in more than a four-fold increase in the population protected by comprehensive smoke-free legislation since 2007 [6]. Despite this progress, less than 20% of the world population are protected from SHS [2, 6, 8], which highlights the need for more extensive adoption and implementation of FCTC Article 8.

Among countries that have adopted a comprehensive smoke-free law, there is variation in the extent to which the law has been successfully implemented, with some jurisdictions such as Ireland and New Zealand reporting high compliance soon after implementation [9], and others – such as Mexico, Uruguay and Kenya - where compliance was initially lower [10, 11]. Although the WHO and other agencies have published guidelines to support the implementation of FCTC Article 8 [9, 12], there is limited evidence regarding the way in which smoke-free laws have been implemented in LMICs.

By 2014, only six African countries had enacted a national comprehensive smoke-free law [13]. Thus, when Uganda’s Tobacco Control Act 2015 [14] (hereafter referred to as ‘the Act’) came into effect in May 2016, Uganda became one of the few African countries to enact a comprehensive national smoke-free law. The Act prohibits smoking within 50 m of all public places, including workplaces, transport terminals and other outdoor spaces. Individuals responsible for public spaces must display clear, prominent no-smoking signage in local languages (including English and Swahili). The Act prohibits the importation, sale and distribution of electronic nicotine delivery systems, shisha (flavoured tobacco inhaled through a pipe) and smokeless tobacco. Potential consequences for non-compliance include penalty fines, business closure for six months, or imprisonment. The provisions of the Act are enforced by authorised enforcement officers, who include Public Health Officers, National Environment Management Authority Inspectors, Police, Customs Officers and any other individuals appointed by the Minister of Health [14].

Prior to the Act, exposure to SHS was known to be especially high at hospitality venues; in 2013, an estimated 62% of adults in Uganda (61% of non-smokers) were exposed to SHS in bars and nightclubs and 16% (16% non-smokers) in restaurants [15]. WHO guidelines state: “civil society has a central role in building support for and ensuring compliance with smoke-free measures, and should be included as an active partner in the process of developing, implementing and enforcing legislation” [16]. Since Civil Society Organisations (CSOs) are potentially well placed to support implementation, we assessed CSO perceptions of the process of Uganda’s new smoke-free law implementation, and the challenges and opportunities in regards to achieving compliance, with a particular focus on hospitality venues.

## Methods

### Sample

We used a purposive sampling strategy, which allows for the collection of in-depth data from a small number of “information-rich” individuals [17]. This approach yields detailed insights and understandings (as opposed to quantitative generalisations). A co-author (Kellen Nyamurungi; KN) identified CSOs based in Kampala that were actively involved in tobacco control as at August 2016; individuals in advocacy or management roles within those organisations were invited to participate in a face-to-face interview. We collected data three months post-implementation to ensure participants had adequate recall of implementation activities and challenges. We focussed on CSOs because their involvement in policy implementation is imperative and strongly endorsed by WHO [16].

### Qualitative approach

We used qualitative description, a pragmatic qualitative research method with an emphasis on obtaining information for practical application [18]. Qualitative description provides a “rich, straight description” of the data, as opposed to a highly interpretive meaning of an experience, or theory development [18]. Qualitative description employs generic qualitative methods, including interviews, reflection on the interviews, coding data into themes and analysis [19]. We used a semi-structured interview guide, whereby discussion topics were specified in advance, though flexibility in wording and sequencing of questions was retained by the researcher to ensure the interview remained natural and conversational [17]. The interview guide consisted of introductory questions about the nature of the new legislation, and subsequently probed participants’ perceptions about the level of compliance in hospitality venues, the implementation activities that had taken place, the barriers and challenges in relation to implementing the law, potential strategies to improve compliance, and the role of CSOs.

The study protocol was approved by the Ethics Committee at Makarere University School of Public Health and registered with the Uganda National Council for Science and Technology. Written informed consent was obtained from each participant, and participants were advised the results would be published. Interviews were conducted face-to-face in English by trained Research Assistants and were audio-recorded and transcribed for analysis. Coding was completed by the lead author using NVivo software. Co-author KN reviewed the transcripts, and the key themes were agreed on after discussion between the lead author and KN.

## Results

Fourteen respondents agreed to participate in an interview. As there are a relatively small number of individuals working for CSOs in Kampala we chose to protect study participants’ identities. Participants typically held leadership positions; their roles included Executive Directors, Program Directors, Founders and Advocacy Officers, and their tobacco control activities included advocacy, research, health education, awareness raising, school-based preventative health, and community mobilisation. Organisations were involved either specifically in tobacco control, child and youth health, or public health/non-communicable disease programmes generally. Interviews lasted on average 40–45 min.

### Contributing factors to low compliance

#### Perceptions of compliance

All participants reported the level of compliance in bars and restaurants was very low, with smoking still occurring regularly in these venues:

*“I don’t want to say that people have even started complying, businesses are continuing as usual.” (Key Informant; KI-10)*

Many bars and restaurants reportedly continued allowing designated smoking areas on-site, which may reflect either a lack of understanding of the law, or a deliberate strategy to undermine the law:

*“…actually most of the bars that I have been to, there is a… smoking zone. Yet in the law, it actually bans or forbids public places from having free smoking zones. They must go 50 metres away from the public place, so that element alone is still lacking…” (KI-12)*

The second main aspect regarding non-compliance was the continued widespread use of shisha at venues, despite the new law making this product illegal. Participants reported that proprietors sold shisha as a marketing strategy to attract young customers to their establishment:

“…most of the bars are flooded by young people within the age bracket that I have been talking about, the below 30s… this person is buying a bottle of shisha at around 15000-20000* at the end of the day, that is big business, they just let the people smoke, so the bars haven’t been compliant.” (KI-3) *equivalent $4.20-$5.60 USD

Two participants insinuated that it was too early in the process of law implementation to expect compliance and hoped that compliance would increase post-enforcement. However, this was a minority view, and most participants expressed the need for activities to enhance law implementation.

#### Inadequate government implementation

A common suggestion was that the government of Uganda tended to be poor at implementing and enforcing legislation. A history of poor implementation was reported to have implications for the smoke-free law, because there was little motivation for businesses to comply:

*“..it has not been implemented yet, and the people we have shared with don’t think it will be implemented, so they are not complying… people are just taking advantage by the fact that it has not been done.” (KI-9)*

Specific challenges identified by participants were that the government had not established a governing body responsible for enforcement nor had they finalised regulations associated with the law. Thus, there had been delays with establishing enforcement processes and structures:

*“..the other challenge is that the regulations and the development of enforcement process is delayed, and yet some of the activities cannot kick off until the regulations have been adopted. Another one related to that is that the law control committee is not yet in place, and the process to nominate members of the committee is delaying, I think those are the key or main challenges we have.” (KI-5)*

The delays and lack of regulations were said to have affected implementation activities, and participants described a lack of consistent signage for hospitality venues (and other public places) to inform the public about the smoke-free law:

*“…there is a regulation that talks about the smoke free signage, so the specifications should come from Ministry of Health, but the actual printing is going to be done by the owners of the public places, and the regulations are going to specify where those signages should be placed… You cannot expect people just to place the signage when you have not told them about it, so they need to know that the law requires them to place the signage and they need to know the penalty for not doing that.” (KI-5)*

An additional problem was the slow pace of action in the sector in contrast to the level of discussion about the problem:

*“One problem with Uganda is that we do not actionalize our things. Many workshops, but, as I speak now so many people are in workshops, so many people are in conferences but we do not actionalize. We leave things on the table.” (KI-1)*

#### Lack of awareness and enforcement

Because of poor implementation, most participants reported a widespread lack of knowledge about the smoke-free law among the public, hospitality venue proprietors, and even implementation agencies:

*“..one of the challenges is that implementing agencies themselves have not really understood what the law is about, and it is even worse to the public.” (KI-7)*

Enforcement was reported as minimal, and two participants cited lack of capacity and low knowledge of the law as principal reasons. One participant alluded to bribery between hospitality venue owners and enforcement agencies:

*“…our enforcement bodies probably are under-staffed, under-motivated, under-paid… Because they are not motivated, they are not educated, they may easily be paid off by the bar owners.” (KI-11)*

Better education of the enforcement agencies and stronger government leadership may prevent bribery.

#### Tobacco industry interference

One of the main challenges undermining compliance was said to be interference from the tobacco industry, and misinformation about the detrimental impact of the smoke-free law on businesses:

*“…we have an enemy that is against the law, and makes the intention of the law to delay, and that is the tobacco industry. The tobacco industry is deliberately misleading the public, and these owners and managers of public places, and part of the public… they have been given misleading information about the law…” (KI-5)*

Participants reported that relationships between tobacco manufacturers and the government were a barrier to full law implementation:

*“…some of them contribute big tax to the Government, so the Government might be compromising in one way or the other.” (KI-2)*

### Opportunities for increasing compliance

#### Raising awareness

The most common suggestion for improving compliance in bars and restaurants was increasing awareness and knowledge of the smoke-free law among the public, and via targeted education aimed at hospitality venue proprietors. Participants believed there was an important role for CSOs in helping to implement the law, and that this role could involve education and stakeholder engagement:

*“We should have a deliberate effort to reach out to… owners and managers of public places, so that we impart knowledge about the Act and tell them what their role is in enforcement of this law. We could probably develop some simplified materials for public place owners and managers, and then distribute them to those places but also talk to them and hear the challenge they are facing so far… And I think we can do this hand in hand with the enforcement agencies…” (KI-5)*

It was suggested that education needed to include the health risks of smoking, details of the law (e.g. the 50 m stipulation, signage requirements, penalties, that shisha is illegal), roles and responsibilities of venue staff, and evidence to dispel the myth that hospitality businesses would suffer if they became smoke-free:

*“I have heard talk of not having shisha or not having tobacco around our premises lowering the pleasure or lowering the number of people who will come, which is not true. There is enough research all over the world to educate, but we need to bring this to the attention of these people. They also must know the consequences for non-compliance…” (KI-6)*

To help hospitality workers, participants suggested monthly meetings, workshops, engaging hospitality employee organisations, helping business proprietors map out action plans for a smoke-free policy, and developing simple, visual educational tools for bars and restaurants.

Public awareness was also seen as a crucial step before compliance could be achieved. A range of different educational strategies was identified, and radio was suggested as the best medium to reach the general public, due to limited literacy. Disseminating messages into communities via local leaders (e.g. church and mosque leaders and local political leaders) was also suggested as an effective method of education:

*“First, the messages should be simplified, and then they have to be translated to the local languages because most people are either illiterate or semi-literate, there are those who cannot read. And then in terms of channels I think the radio will be better because almost all part of this country at least they listen to radio stations and a bit of person to person is necessary. The community Balazas [community meetings] where those implementing the law can meet community members and talk to them about the law, I think it can be effective...” (KI-5)*

Other means of communicating with the public included television, billboards and social media, the latter which may only reach younger, more educated, urban populations.

#### Enhancing enforcement

Although increasing awareness of the law was seen as the first step, participants offered further suggestions on how to enhance enforcement. Enforcement agencies themselves were identified as being in need of information and education about the law:

*“..we need to bring the people who are going to help in enforcing these laws on board, through awareness and education. And one of them of course is police, we need really to train police on this particular law, and make them aware.” (KI-1)*

Key elements of effective enforcement included surveillance and regular visits by authorised enforcement officers to venues to warn hospitality staff that the law would be enforced:

*“…the moment you talk to them, to the manager, you need a follow up. So you need to make a follow up about such things otherwise people take things for granted… Notification needs to be taken on a frequent basis.” (KI-12)*

Other suggestions included the enactment of penalties (e.g. fines, suspension of licence) for breaches of the legislation, which had not yet been occurring, and publicity of penalties as a deterrent for future breaches.

#### Civil society roles

Overall, civil society groups considered themselves as having a key role to play in educating the public and key stakeholder groups:

*“Awareness creation, sensitization, and dissemination, we are civil societies and our role is clearly outlined, our job as advocates is to make as much noise about this issue...” (KI-3)*

One role that civil society groups could take included advocacy for the enactment of penalties and for tougher penalties if compliance remained low:

*“..perhaps we could either lobby or advocate for [the] consequence to be a bit tougher or more threatening to the bar owners. Because, however much some people you educate them, they may not be affected… If the law is more threatening to the bar owners and businesses, we could advocate for that.” (KI-11)*

Participants identified a range of support that they felt was needed for their organisation and the wider sector to achieve better compliance. These included financial resources for education and dissemination, research and data about the harms of tobacco, capacity building for people and groups engaged in tobacco control, and better partnerships and coordination with other agencies and other sectors, including the media.

## Discussion

This qualitative research provides evidence of the difficulties encountered when a country adopts and implements a comprehensive smoke-free policy and the factors that inhibit successful implementation and compliance. It suggests firstly, that Uganda’s government may not have adequately planned for law implementation, and that the necessary structural changes (e.g. a governing body for enforcement and finalised regulations) were not in place three months post-implementation. Secondly, it suggests the tobacco industry has possibly undermined implementation through spreading misinformation. This research also identifies two main areas of non-compliance specific to Uganda; the sale and use of shisha at hospitality venues, and the ongoing presence of designated smoking areas. The latter could indicate either a lack of adequate information about the law, or a deliberate attempt by hospitality venues to subvert the law in the absence of enforcement. Consistent with WHO recommendations, strategies for enhancing compliance were identified as awareness-raising, education, training, and strong enforcement [12].

There is wide variation in the extent to which smoke-free laws have been effectively implemented [9]. Examples from Ireland and New Zealand, which enacted national smoke-free legislation in 2004, suggest intensive educational campaigns, pervasive compliance checks, and a complaints system to enable the public to report violations, contributed to high compliance rates soon after implementation [9, 12]. Although there is limited available research, evidence suggests LMICs tend to have lower compliance compared to high-income countries [10, 11, 20]. In 2006, Uruguay became the first middle-income country to pass a comprehensive smoke-free law [9]. Studies by the International Tobacco Control Project in 2008 and 2010 suggest non-compliance ranged from 6 to 9% in restaurants, and 8–36% at bars in Uruguay, leading the researchers to recommend new efforts and strategies to enhance compliance [11]. One exception to lower compliance in LMICs is the Seychelles where, nine months post-implementation, compliance was very high at bars and restaurants, with an observational study reporting no visible smoking in venues [21]. Factors likely to have contributed to high compliance include the small country size (requiring fewer resources for implementation), high awareness and knowledge of the smoking ban among hospitality staff, training of hospitality staff on how to enforce the ban, and active enforcement of the ban by venue workers [21].

The extent of community awareness of a smoke-free law influences the level of compliance [9]. Thus, in the period preceding law implementation, comprehensive education should be delivered to help the general public and business owners understand the purpose and the implications of the law [8, 12]. WHO guidelines for implementing Article 8 highlight the importance of advanced planning, resource mobilisation, monitoring (of compliance and tobacco industry activities) and policy evaluation [12, 16]. Strong enforcement is crucial, and is particularly important immediately after implementation until high levels of compliance are reached, after which a policy becomes self-enforcing [8, 12].

Many LMICs have passed *partial* smoke-free laws, which include smoke-free bars and restaurants as areas accessible to the public where smoking is prohibited (as opposed to comprehensive laws prohibiting smoking in *all* public places). Even this approach can result in low compliance in bars and restaurants [10], which suggests compliance in these venues may be particularly challenging. The tobacco industry may place more strategic importance on undermining smoke-free laws at hospitality venues than compared to other places, given the well-established link between alcohol consumption and smoking [22]. It is possible that low-income countries need additional support over and above initiatives such as WHO guidelines, to enable them to effectively plan for policy implementation and resist industry interference. Examples may include additional funding and capacity-building programmes to strengthen the CSO sector, and further guidance and resources from WHO to enhance planning, coordination and overall implementation. This is crucial because, as demonstrated in the Netherlands, even after a comprehensive smoke-free law has been passed, tobacco industry efforts to undermine compliance in hospitality venues can result in policy reversal [23]. Article 5.3 of the FCTC offers governments a set of strategies to protect public health against tobacco industry interference, but its guidelines tend to be underutilised [24]. Experts argue a crucial first step in addressing industry interference is changing attitudes towards the tobacco industry, by actively monitoring and exposing its conduct [24].

By 2014, only six African countries had implemented a national comprehensive smoke-free law [13], which is likely to increase steadily as the tobacco control sector responds to industry attempts to exploit LMIC markets. Uganda’s failure to adequately plan and finance law implementation in a timely manner serves as an important lesson to other LMICs that may implement similar laws. A review focusing on the implementation of smoke-free laws in Africa suggests that policymakers frequently underestimate the political complexity of adopting, implementing and enforcing such laws [25]. One of the key factors that dictated whether a smoke-free policy was implemented effectively was the presence of a strong tobacco control CSO movement [25], which further highlights the important potential offered by CSOs as partners in smoke-free law implementation.

Our study has limitations. The sample may have comprised people particularly motivated to talk about the smoke-free legislation. However, since many participants expressed consistent views about low compliance and strategies to enhance compliance, it is unlikely that the sample was biased in this regard. It could be argued that collecting data three months after implementation did not allow sufficient time for venue owners to transition to the new law; however, our study was primarily interested in the process of law implementation rather than on assessing the extent of compliance, which has been reported elsewhere [26]. Our findings are not necessarily transferable to other LMICs, nor indeed to other areas of Uganda. A limitation with all qualitative research is that the views and beliefs of the researchers may influence the study process, from conceptualisation and data interpretation [27]. However, our data were collected by three Research Assistants who were independent of the research team, which reduced the likelihood that interviewer bias or assumptions affected the data collection process. Given our team’s public health expertise, it is plausible that researchers from different disciplines (e.g. law or economics) would offer different perspectives on Uganda’s experience of smoke-free law implementation. A strength of the in-depth interview approach is that it provides detailed insights into CSO experiences and viewpoints, and the factors underpinning poor compliance.

## Conclusions

Given the time and effort required before governments adopt comprehensive smoke-free legislation, poor implementation following law enactment is a missed opportunity for improving and sustaining public health. Our study suggests there is potential for CSOs to support the implementation of the smoke-free law in Uganda through public awareness-raising, targeted education for hospitality staff, and advocacy for better enforcement. The government could coordinate activities to realise the potential offered by CSOs and address the issues that these groups have identified as impeding smoke-free hospitality venues.

## Abbreviations

CSO: Civil Society Organization
FCTC: Framework Convention on Tobacco Control
KI: Key informant (i.e. study participant)
KN: Kellen Nyamurungi (a co-author of this study)
LMICs: Low and middle-income countries
SHS: Second-hand smoke
the Act: Uganda’s Tobacco Control Act 2015
WHO: World Health Organization
